# Genomic determinants, architecture, and constraints in drought-related traits in *Corymbia calophylla*

**DOI:** 10.1186/s12864-024-10531-8

**Published:** 2024-06-27

**Authors:** Collin W. Ahrens, Kevin Murray, Richard A. Mazanec, Scott Ferguson, Ashley Jones, David T. Tissue, Margaret Byrne, Justin O. Borevitz, Paul D. Rymer

**Affiliations:** 1 Hawkesbury Institute for the Environment, Western Sydney University, Richmond, NSW 2753 Australia; 2 Cesar Australia, Brunswick, VIC 3058 Australia; 3 Research School of Biology, Australian National University, Canberra, ACT 2600 Australia; 4 Biodiversity and Conservation Science, Western Australian Department of Biodiversity, Conservation and Attractions, Kensington, WA 6151 Australia

**Keywords:** Eucalyptus, Epistasis, Pleiotropy, Genome wide association study (GWAS), Water use efficiency, Heritability

## Abstract

**Background:**

Drought adaptation is critical to many tree species persisting under climate change, however our knowledge of the genetic basis for trees to adapt to drought is limited. This knowledge gap impedes our fundamental understanding of drought response and application to forest production and conservation. To improve our understanding of the genomic determinants, architecture, and trait constraints, we assembled a reference genome and detected ~ 6.5 M variants in 432 phenotyped individuals for the foundational tree *Corymbia calophylla*.

**Results:**

We found 273 genomic variants determining traits with moderate heritability (*h*^2^_SNP_ = 0.26–0.64). Significant variants were predominantly in gene regulatory elements distributed among several haplotype blocks across all chromosomes. Furthermore, traits were constrained by frequent epistatic and pleiotropic interactions.

**Conclusions:**

Our results on the genetic basis for drought traits in *Corymbia calophylla* have several implications for the ability to adapt to climate change: (1) drought related traits are controlled by complex genomic architectures with large haplotypes, epistatic, and pleiotropic interactions; (2) the most significant variants determining drought related traits occurred in regulatory regions; and (3) models incorporating epistatic interactions increase trait predictions. Our findings indicate that despite moderate heritability drought traits are likely constrained by complex genomic architecture potentially limiting trees response to climate change.

**Supplementary Information:**

The online version contains supplementary material available at 10.1186/s12864-024-10531-8.

## Introduction

Climate change is increasing the intensity and frequency of droughts worldwide [1], pushing trees to their physiological limits, and in some cases to the point of failure, resulting in forest dieback [2]. A species’s ability to tolerate drought is likely determined by complex genome characteristics, including base pair changes [3, 4], large rearrangements [5], and/or interactions between genes [6, 7]. Understanding the genetic mechanisms that control drought related traits can lead to better predictions of drought tolerance, increasing our success in managing natural and planted forests under climate change induced drought.

Droughts are major selective forces [8, 9], however it is generally unknown how much variation of drought tolerant traits in trees are genetically controlled. There are many traits that provide tolerance to drought conditions. One drought trait that stands out is carbon isotope discrimination or the ratio between C13 and C12 (δ^13^C). Isotope discrimination is important because it is based on Rubisco’s preference for light carbon (C12), and plants with a higher proportion of C13 are generally more drought tolerant [10]. Further, δ^13^C has been strongly correlated to stomatal conductance [11] and water use efficiency [12]. Most studies that link genotype to drought tolerance (as δ^13^C) have been performed on agriculturally important species, such as a study that shows some evidence of genetic control of δ^13^C in soybeans [13]. However, a recent study on an ecologically important species identified 78 and 6 drought tolerant variants related to δ^13^C in coast redwoods and giant sequoias, respectively [14], and another study used a few thousand single nucleotide polymorphisms (SNPs) for *Q*_ST_–*F*_ST_ comparisons to identify selection occurring for δ^13^C in *Pinus pinaster* [15]. A second often used trait that is associated with drought tolerance is specific leaf area (SLA), which is the leaf surface area per unit of dry biomass [16, 17]. A study on *Populus trichocarpa* found two SNPs associated with this trait [18]. However, other studies suggest that SLA is highly plastic and largely not heritable [19, 20]. A third trait used to quantify the effects of drought is the normalized difference vegetation index (NDVI) which measures chlorophyll reflectance [21]. A study on maize found nine potential adaptive SNPs controlling NDVI [22]. Natural selection acts directly upon expressed phenotypes [23], which are controlled by additive and non-additive genetic variation [24]. Most studies linking genotype and phenotype using GWAS focus on quantifying the additive genetic variation controlling the trait of interest [24, 25]. However, by explicitly understanding the contribution of both additive and non-additive genetic variation to drought tolerant traits, we could improve predictions on how well populations can respond to novel drought conditions.

It is inherently difficult to quantify the genotypic effect on physiological traits when measuring plants in situ because the variation could be due to environment and not genotype [26]. Growing related individuals from many populations in a common garden minimises the environmental variance [27] resulting in the phenotypic variance being the product of the genotypic differences. This allows for the estimation of trait heritability, which can be interpreted as part of the trait’s ‘evolvability’ or evolutionary potential [28]. Common gardens are also an important resource to use in conjunction with genome-wide association studies (GWAS), which explicitly evaluates each correlation between SNP and trait using mixed effects linear models. GWAS studies have been deployed for a vast number of species to understand the genetic determination of trait variation and gene discovery [29–31]. Therefore, GWAS is a powerful technique that allows for the identification of within and among population standing genetic variation related to complex traits.

Estimates of trait heritability can be determined based on genetic variants (i.e., SNPs) contributing to phenotypic variation in GWAS analyses using an additive genetic framework. However, heritability from GWAS analyses is unable to account for non-additive factors that could contribute to the heritability of a trait. Missing heritability in complex traits may be due to non-additive genetic variation such as gene–gene interactions (epistasis [32]) and gene-trait interactions (pleiotropy [33]). Accounting for epistatic and pleiotropic interactions that contribute to the heritability of quantitative traits can improve model predictions [33, 34]. Epistatic effects could enhance or remove the effect of a gene on the trait depending on the presence of interacting genes in the genetic background. The widespread presence of epistasis could limit our ability to identify all the heritable variation but could constrain or boost adaptation of traits. Another biological process that could potentially constrain traits is pleiotropy because causal genes could be potentially interacting with multiple traits [35]. Pleiotropy could result in an antagonistic behaviour where one gene positively affects one trait while negatively affecting a second trait [36]. Together, epistasis and pleiotropy can impose significant constraints for adaptive traits, however they are not often quantified limiting understanding of how organisms may respond to their environment.

Our purpose for this study was to investigate the genetic determinants of drought related traits and their relationship to one another in an ecologically, economically and culturally important tree species. We used over 6 million SNPs and phenotyped 432 trees under common garden conditions for three drought-related traits to identify SNPs related to drought; we investigated trait heritability, genomic architecture, functional annotation, and gene interactions between multiple traits. We hypothesise that genomic architecture (epistasis and haplotype blocks) plays an important role in determining traits and that pleiotropy may constrain other drought-related physiological traits. We discover several significant genes, overabundance of significant loci in *cis*-regulatory regions, and many epistatic and pleiotropic interactions between significant SNPs that may constrain drought traits in a foundational Australian tree species, *Corymbia calophylla*. These complex genomic architectures are likely to play important roles when managing natural and planted forests for drought under climate change.

## Results and discussion

### Species and functional traits

**Fig. 1:**
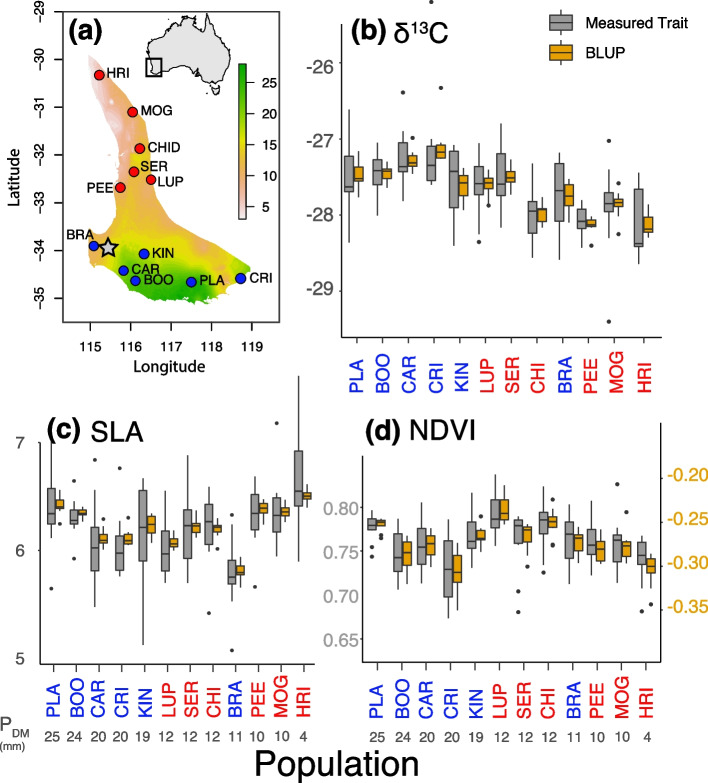
Phenotypic traits were studied in a common garden of populations sampled from across the range of *Corymbia calophylla*, denoted here by the precipitation layer. **a** Location of populations sampled and the experimental site mapped with precipitation of the driest month (P_DM_; mm; BIO14); **b**-**d** trait values (grey) with their best linear unbiased predictions (BLUPs; yellow) for (**b**) δ^13^C, (**c**) SLA, and (**d**) NDVI. Population colours are coded red for northern populations and blue for southern populations and ordered from wettest (left) to driest (right). Star represents the location of the experimental site. Inset shows location of study area within Australia. NDVI was scaled (y2-axis; yellow) to meet assumptions of normality before estimating BLUPs

We quantified variation in three functional traits indicative of drought resistance (δ^13^C, NDVI, SLA; Fig. 1b-d). Two of the three focal traits (δ^13^C: *h*^2^ = 0.17**; NDVI: *h*^2^ = 0.15**; SLA *h*^2^ = 0.08) exhibited small, but significant, narrow-sense heritabilities based on quantitative genetic models [29], indicating genetic determination by polygenic mechanisms. We controlled for random variation of the measured trait values using best linear unbiased predictions (BLUPs) and found that the three traits varied across families and populations (Fig. 1b-d). A linear model showed that traits were significantly differentiated among populations (F_11,419_ = 88.89; *p* < 0.001) and among families within populations (F_23,407_ = 53.75; *p* < 0.001).

### Draft genome and genome-wide association studies (GWAS)

We assembled a high-quality de novo genome (350 Mb haploid size, 100 contigs, contig N50 = 3 Mb, 11 pseudochromosomes, NCBI accession #: GCA_014182845.1). We then aligned individual short read sequences and identified 91 M pre-filtered single nucleotide polymorphisms (SNPs) (Figure S1; Table S1). First, low quality samples were removed if their missingness was below 0.5. Then variants were filtered on quality (Phred > 25), minimum read depth (6), missingness (max = 0.2), and allele frequency (minor allele frequency = 0.01), 6.49 million informative SNPs were discovered, averaging a SNP every 60 bases. Linkage disequilibrium (LD) decayed quickly, as median base pair distance to half-maximal *r*^2^ values were 160 base pairs (Table S2). We also estimated the LD scores across each chromosome (Figure S2) and similar LD patterns for each chromosome. These mean LD estimate (160 bp) is greater than previous half-maximal estimates of LD decay in *Eucalyptus* species (92 and 113 bp) [39], confirming that there is a very high degree of population diversity and recombination in the system.

**Fig. 2:**
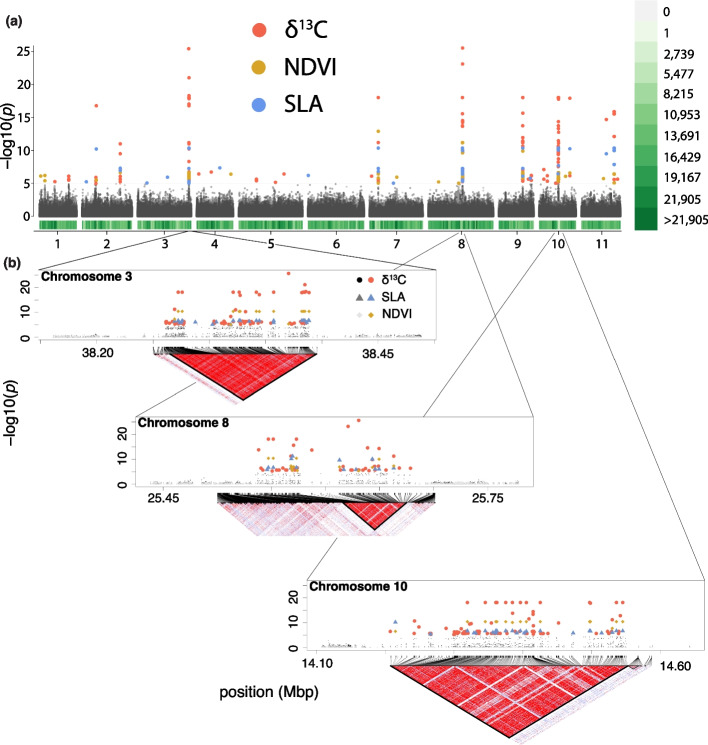
Genome sampling and GWAS outputs for *Corymbia calophylla*. **a** Manhattan plots for three traits and SNP density. Points represent SNPs significantly associated with the trait (red = δ^13^C; yellow = NDVI; blue = SLA; grey = not significant) at an FDR value < 0.00001. The density plot (below Manhattan plot) shows the number of SNPs in 1 million base pair segments across the genome with colour (white to green). **b** Magnified view of the significant peaks within the three ‘hotspot’ regions of adaptive variation. Underneath the magnified views are haploview plots that detect significant blocks, bounded by black lines. The linkage disequilibrium (r.^2^) across the haploview plot is denoted from low (white) to high (red)

To discover SNP-trait associations, we performed genome wide association studies for the three functional traits. Weak but detectable population structure (*F*_ST_ = 0.05) was controlled for in the GWAS analysis using the first 10 axes of a multidimensional scaling plot, the first two axes show a distinction between the northern and southern populations (Figure S3). The resulting GWAS identified 279, 69 and 92 significant SNPs for δ^13^C, SLA, and NDVI, respectively (Fig. 2a). Candidate SNPs were found on all chromosomes across the genome with several regions having a high density of candidate SNPs with peaks on chromosomes 3, 8, and 10 (Fig. 2a). Magnification of these peaks highlights many SNPs that occur in large haplotype blocks (150–350 kb) based on Haploview results, beyond the median LD decay, interspersed with non-significant SNPs, and different among all three traits (Fig. 2b). These patterns within gene-rich regions could be due to structural variants such as inversions [40] or large haplotype blocks, which have been found to be important in adaptation in other systems (sunflower ecotypes [41] and teosinte [42]). Structural variants are a significant source of variation contributing to adaptation [43], are non-randomly distributed throughout the genome [44], and can change gene expression patterns [45]. The patterns found, particularly the association of trait-associated SNPs with significant haploblocks, in our study could be due to structural variants, but long-read sequencing would have to be performed for confirmation. Large haplotype blocks likely explain the strong LD between candidate SNPs within chromosomes (Figure S4d-f) with significant LD for the haploblocks in chromosome 3 (*r*^2^ = 0.35; *p* = 0.03), chromosome 8 (*r*^2^ = 0.25; *p* = 0.02) and chromosome 10 (*r*^2^ = 0.29; *p* = 0.04) associated with δ^13^C. However, these patterns do not explain any of the long-range LD across chromosomes (Figure S4a-c). The mean r^2^ across all significant SNPs associated with δ^13^C is 0.28 with a mean *p*-value of 0.04. This is likely due to rarity disequilibrium (i.e., genetic indistinguishability – giSNP [46]), which is a widespread phenomenon that arises when interchromosomal SNP pairs are in perfect LD due to the combinatorial limit on unique genotype patterns in finite sample sizes and may be contributing to the pattern. Even though we do not know the genomic mechanism of these haplotype blocks, we can be confident that the target genes within regions are indicative of the genetic architecture associated with quantitative traits due to high significance and extremely high LD between significant SNPs, and given the pattern persists after removal of the giSNPs.

To determine how much trait variation could be explained by all genomic SNPs, we estimated the SNP-based heritability to explain the total proportion of variance in phenotypes [47]. We found that SNP-based heritability for all three traits (δ^13^C *h*^2^_SNP_ = 0.55 (SE 0.14); SLA *h*^2^_SNP_ = 0.27 (0.12); and NDVI *h*^2^_SNP_ = 0.66 (0.14)) was much greater than the heritabilities calculated through quantitative genetics methods (*h*^2^ = 0.11 (0.08), 0.08 (0.08), and 0.15 (0.08) for δ^13^C, SLA, and NDVI, respectively [20]), and genetic correlations (r_g_) depended upon the traits. r_g_ was significant between SLA and NDVI (*p* = 0.008) but not significant between δ^13^C and the other two traits (SLA: *p* = 0.16; NDVI: *p* = 0.55). Even though both NDVI and SLA are weakly correlated wtih δ^13^C at the trait level (SLA: *r*^2^ = -0.2; *p* < 0.001; NDVI: *r*^2^ = 0.19; *p* < 0.001), both correlative patterns disappear at the genetic level. This paradox could indicate that correlational selection could be occurring among traits [48] or alternatively there is a complex system of gene reuse among traits that are difficult to detect [49]. Indeed, complex evolutionary patterns have been observed in eucalypts where the same gene is reused in diverging ways under the same selection environments [50]. There were also major differences between the three *h*^2^_SNP_ estimates. Considering the large *h*^2^_SNP_ for both δ^13^C and NDVI, which includes all SNPs, we also identified several SNPs with large effect sizes (top SNP for δ^13^C 0.25, SLA 0.34, NDVI 0.17; Table S3). Theoretical work indicates that in highly polygenic traits, alleles with very small effect sizes could be ephemeral because they are prone to swamping by gene flow as different genotypic combinations can provide optimum fitness [51]. While these ephemeral, swamping prone SNPs, could contribute to our traits, they would be impossible to differentiate between neutral alleles in a GWAS framework. On the other side of the effect-size spectrum, the top 10 candidate SNPs associated with δ^13^C showed greater effects (18—25%) (Table S3; giSNPs were dropped from this model), compared to the greatest explanatory SNPs for NDVI and eight of the top 10 SNPs for SLA. These ten SNPs accounted for ~ 50% of the variation for δ^13^C and SLA, and 34% of the variation in NDVI. The lower combined *r*^2^ value for NDVI compared to the other two traits might be due to many factors, including more trait variation, more SNPs of small effect, and more epistatic interactions. The inclusion of epistatic interactions increased the variation explained for δ^13^C and NDVI, but slightly lowered the variation explained for SLA, such that epistatic interactions improve phenotypic predictions for two of the three traits compared to individual SNP effects.

There is also a proportion of trait variation that was not explained. One explanation might be that these traits are highly polygenic with very small additive effects, and we were only able to identify the variants most strongly associated with the phenotype [52]. *h*^2^_SNP_ should capture these undetected small effects, but this only explained a quarter to two thirds of the variation in our drought traits. Another explanation is that structural variants, which were not included in this dataset, could explain some of the missing heritability as they are known to be ubiquitous and have the potential to explain a large proportion of heritable genetic variation [55]. Even though we performed our experiment in a common garden to minimse the effect of the environment, phenotypic plasticity could still play a role in these trait differences through variation in gene methylation or control through regulatory elements among genotypes sourced from different environments [26, 53, 54]. Methylation could result in non-heritable differences, while variants in regulatory elements could result in differences in plastic responses that are heritable. We detected genomic variants in the regulatory region (details below), which explained a large proportion of the trait variation, despite complex interactions constraining adaptation, and provide insights into the adaptive role of genes and regulatory elements.

**Fig. 3:**
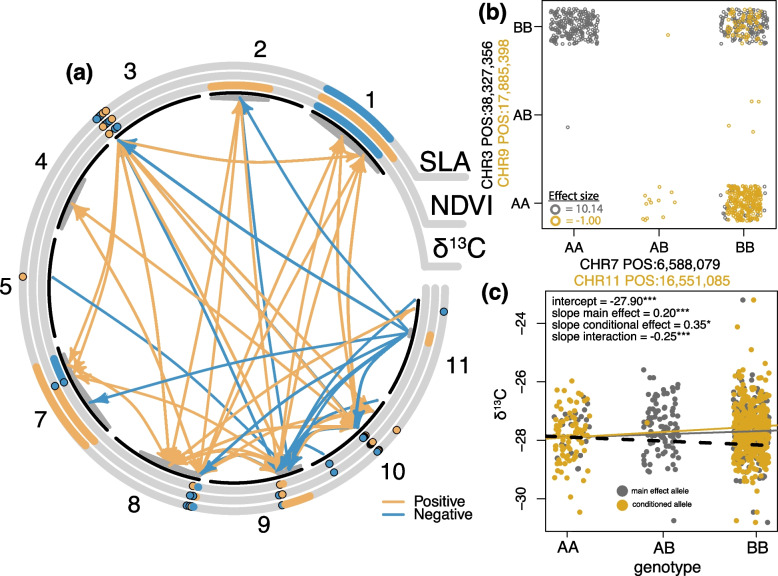
Patterns of significant epistatic and pleiotropic interactions in *Corymbia calophylla*. **a** Epistatic interactions are shown with coloured arrows and pleiotropic effects between traits are shown in the circular bands. Chromosomes are in black; chromosome six is not present because no SNPs were significant in the analysis. The direction of influence is shown by colour, where orange indicates that the SNP affects a different SNP in a positive way and blue is indicative of a negative effect. Interactions between a SNP and multiple traits indicate pleiotropy, while the same colours are indicative of the same effects. Antagonistic pleiotropy is inferred if the colours are different among SNPs in the same chromosomal location. Points on chromosome 3, 8, and 10 have been manually separated due to severe overlapping to visualise the antagonistic effects. **b** Genotypes for negatively influenced epistatic interaction between two SNP variants (grey points in (**b**) & blue arrows in (**a**)) and positively influenced epistatic interactions between two SNP variants (orange points in (**b**) & orange arrows in (**a**)). **c** Visualisation of one significant epistatic interaction where the main effect of a SNP (grey in (**c**)) and trait is conditioned on a second SNP (yellow in (**c**)), black dashed line is the interaction effect between the two variants. *P*-value – *** < 0.001; * < 0.05

Complex gene and trait interactions (i.e., epistasis and pleiotropy) are known to play important roles in quantitative traits [56, 57]. We found evidence that some of the unexplained trait variation could be attributable to epistasis among significant SNPs identified in the GWAS. Gene interactions among the candidate SNPs were explicitly evaluated using CAPE (giSNPs are removed from this analysis), revealing significant epistatic interactions across the genome (*p* < 0.05; Fig. 3a), with strong interactions between chromosomes 3, 8, 9, and 10. Main effects between two SNP pairs are shown between chromosomes 3 & 7 (negative effect; blue arrows in 3a) and 9 & 11 (positive effect; yellow arrows in 3a) (Fig. 3b), and we also provide visualisation of an epistatic interaction when the main effect (variant + trait) is conditioned on a second variant (Fig. 3c), where the interaction between the two SNPs affects the trait in a negative way (Fig. 3c dashed line). We then assessed possible pleiotropic interactions between the three traits, i.e., the effect of one SNP on multiple traits. Pleiotropic interactions are shown in Fig. 3a in the concentric bands, where the same SNP is highlighted for more than one trait. We identified several cases within chromosomes 1, 3, 7, 8, 9, and 10 that were due to pleiotropic effects (Figs. 3a & S3). There was evidence of antagonistic interactions (when a gene affects traits in different directions; represented by different colours along the concentric trait-circles in Figs. 3a & S3). A network plot shows the interactions (both positive and antagonistic) between all 11 chromosomes (Figure S5). Pleiotropy was corroborated through annotation results, for example, there were 11 genes that were found to be significantly associated with all three traits (Table S4). Eight of these genes were expressed during growth and development processes, including in plant organs such as guard cell and leaf structure. Pleiotropy is known to play in integral role among correlated polygenic traits. In fact, a recent study on humans shows that 90% of trait-associated loci overlap with other traits and are mostly involved in the regulation of transcripts [58]. It is also known that pleiotropic loci maintain stronger genetic correlations compared to loci in LD [59]. This suggests that the interplay between pleiotropy, regulatory regions, linkage, and r_g_ is a critical component to tease apart the genetic mechanisms controlling drought tolerance in eucalypts. When assessing epistatic and pleiotropic interactions, it is difficult to determine how these control functional traits as there are tens of thousands of pairwise possibilities in our dataset contributing to the overall pattern of adaptation.

### Annotation

In order to identify location (e.g., *cis* regulatory, genic) and effect (e.g., synonymous, nonsynonymous) of adaptation, snpEFF was used to annotate all SNPs. Annotations for all 6.49 million filtered SNPs reveal many moderate (nonsynonymous) and low (synonymous) effect alleles on protein function with a much smaller proportion of high-effect alleles (Table S5). Each chromosome had similar rates of nonsynonymous and synonymous SNPs (Table S5), except for chromosome 8 with a much higher rate of SNPs upstream and downstream of genes and more than double the number of high-effect SNPs than the other chromosomes (Table S5). We then functionally annotated the candidate SNPs within the three chromosomes with the highest significant peaks show interesting patterns (Chromosomes 3, 8, and 10; red in Fig. 2a). For example, of the 41 SNPs on chromosome 8 associated with δ^13^C, 37 are within gene regulatory regions (< 5,000 base pairs upstream of the gene), while the four remaining SNPs were nonsynonymous with a moderate effect and synonymous with low effects (Table 1). We should be cautious in extrapolating this further because of the non-independence between significant SNPs identified in the GWAS analysis within a haplotype block and further work should be performed to identify the causal SNP(s). However, the general overabundance of significant SNPs within regulatory regions compared to genic or intergenic regions is suggestive that regulatory regions play an important role. The regions of adaptive variation in chromosomes 3 and 10 are mostly intergenic with SNPs in gene *cis*-regulatory regions (within 5 kb of a gene on either the 5’ (upstream) or 3’ (downstream) end of the gene) and six candidate SNPs in promoter regions (within 500 bp upstream of a gene) for δ^13^C on chromosome 10. This is notable because sequence variation in regulatory regions differentially impacts the function of nearby genes [60].

**Table 1:** Annotation summary for the candidate SNPs on three chromosomes in *Corymbia calophylla*

Chromosome	Trait	SNPs	Intergenic	Up-reg (5 kb)	Prom (500 bp)	NS	S	Down-reg (5 kb)	Effect (H|M|L)
**3**	SLA	17	14	1	0	0	0	2	0|0|0
	NDVI	23	18	2	0	0	0	3	0|0|0
	δ^13^C	74	59	6	0	1	0	8	0|1|0
**8**	SLA	13	0	13	6	0	0	0	0|0|0
	NDVI	16	0	16	5	0	0	0	0|0|0
	δ^13^C	41	0	37	25	1	3	0	0|1|3
**10**	SLA	16	5	8	0	0	0	3	0|0|0
	NDVI	26	8	11	0	0	0	7	0|0|0
	δ^13^C	88	28	37	6	2	0	19	0|2|0

*up-reg* upstream regulatory region, *down-reg* downstream regulatory region, *prom* promoter region (within 500 bp of a gene), *NS* Nonsynonymous, *S* Synonymous, *H* High effect size (highly disruptive impact on protein function), *M* Moderate effect size (non-synonymous mutations, possible change in protein effectiveness), *L* Low effect size (synonymous mutations, non-coding or intergenic variant), *Intergenic* SNP not found within 5 kb of a gene

There were several significantly associated SNPs enriching genes with functions that provide support for the potential contribution to drought response (Table S6; results from orthofinder and eggNOG). For example, two genes were enriched for lignification and F-box protein (Eucgr.H02869 [61] and Eucgr.H02864 [62] respectively), which are known to support drought tolerance. In addition, the gene Eucgr.D00100 regulates the ethylene hormone, and is an ortholog to the Arabidopsis gene AT4G20880. Ethylene is known to mitigate the negative effects of water and temperature stresses [63]. The Eucgr.D00030 gene is an Ankyrin repeat family protein and has been known to confer tolerance to both drought and salinity in Arabidopsis and Soybean [64]. The possible roles these genes play in drought adaptation for *C. calophylla* will need to be quantitatively verified, but these discoveries provide promising ways in which this species have evolved drought resilience.

### *Cis*-regulatory variants drive trait adaptation

The adaptive variation associated with traits, particularly for δ^13^C, is largely driven by variants in *cis*-regulatory regions (noncoding DNA that regulates neighbouring genes; Table S7 – categorised significantly associated SNPs into four categories 500 bp, 5 kb, 10 kb and 50 kb), which are less constrained by pleiotropy than coding regions from an evolutionary perspective [65]; this mechanism appears to be important in *C. calophylla*. Indeed, recent studies suggest that *cis*-regulatory regions are critical for different types of adaptation [66–68]. Yet there is poor understanding how this variation influences population-level local adaptation, as noted by recent studies on evolution [69]. Here, we characterise variants associated with functional traits that are important for this species’ adaptation to drought that are overrepresented by *cis*-regulatory regions. While we recognise that this finding needs to be confirmed in future research to disentangle non-independence issues within haplotype blocks, our data suggests that adaptation within *cis*-regulatory regions are more abundant than variants found within protein-coding genes and are more likely to shape the genomic architecture of these drought traits. Similarly, recent work has shown that regulatory variants are critical for drought in sunflowers [70]. We currently hypothesise that intraspecific drought-related phenotypes is mostly governed by changes within regulatory regions.

## Conclusion

Considering the impact climate change is having on drought frequency and severity, understanding the molecular underpinnings of drought related traits provides an important step forward in determining the mechanisms controlling drought tolerance. We found heritable genetic variation associated with drought traits within several haplotype blocks across several chromosomes. This is particularly important when considering the abundance of epistatic and pleiotropic interactions, which likely constrain these traits ability to adapt. Furthermore, the majority of significant variants were detected in regulatory regions where they may influence the expression of many genes and traits. Despite the moderate levels of heritable variation determining drought related traits, the complex genomic architecture will complicate adaptive management strategies, i.e., by promoting one trait or gene, other traits or genes may be unexpectedly promoted or suppressed. Using the standing genomic variation in highly admixed natural populations may facilitate adaptation to climate change induced droughts.

## Methods and materials

### Study species

*Corymbia calophylla* is a foundation forest canopy species located in Western Australia (WA). It is considered a foundation species because it is critical for forest structure and ecological processes [71]. *Corymbia calophylla* is an important component of planted forests both for wood production and ecological restoration, provides critical habitat and resources to native animals, as well as having deep connections to the Aboriginal people. This species is an ideal candidate in which to study adaptation of functional traits because its distribution traverses strong environmental gradients over short distances, it has recently experienced mortality events attributed to climate change [37, 72], and evidence of adaptation to climate has been identified in physiological experiments and genome–environment investigations [20, 38, 73, 74].

### Experimental site

This research was conducted in a plantation near Margaret River, WA Australia (Fig. 1 main text), located in the *C. calophylla*’s cool–wet region. Seed collection and trial design have been described in detail elsewhere [38]. Briefly, 18 populations represented by 165 families were established at the experimental site for a total of 3,960 individuals in six replicated blocks with two rows of buffer trees to minimise edge-effects. Seed collections for field trials were performed by Richard Mazanec (WA Department of Biodiversity, Conservation and attractions) and no voucher specimens were collected because the field sites are persistent. Families are defined here as individuals that have a known, common mother but unknown fathers (i.e., half-sibs) via mixed pollination within an intact forest. We focused on 12 populations representing contrasting climate combinations covering the full geographic distribution of *C. calophylla* (Fig. 1 main text). We sampled phenotypes and genotypes from a total of 432 trees, including 4 half-sibs from 10 families within 12 populations when available for a total of 120 families. Permissions for leaf material collection were provided by the land owners and Western Australia’s Department of Biodiversity, Conservation, and Attractions.

### Trait measurements

Traits were measured in March 2017 on *C. calophylla* trees that were 29 months old and 2–3 m tall. For each individual tree, we removed a north facing, mid-canopy side branch at its intersection with the main stem. The side branch was removed in the morning (between 8 a.m. and 12 noon), stored in a cool box, and measured in the afternoon (between 12 noon and 6 p.m.). For each side branch, we collected data for the three traits (among others not listed here): integrated water-use efficiency (δ^13^C), specific leaf area (SLA), and normalized difference vegetation index (NDVI). All traits have shown close association to climate in past studies. High water-use efficiency (WUE) is the link between photosynthesis and evaporation [75] that translates to climatic tolerance under water limitation. Water-use efficiency is correlated with isotope discrimination (δ^13^C, an isotopic signature measuring the ratio of 13C and 12C [76]) and relates to leaf gas exchange properties [77, 78]. To estimate δ^13^C, the leaves were kept in an airtight box with silica gel until they could be dried in an oven at 70 °C for 48 h. δ^13^C was measured from leaves dried using a benchtop freeze dryer (Alpha 1–4 LDplus Laboratory Freeze Dryer, Martin Christ). The leaves were grounded into a fine powder using a cyclotec mill (Foss Analytics) and sent for isotope analysis (ANU Isotope Laboratory) using a coupled EA-MS system (EA 1110 Carlo Erba; Micromass Isochrom).

Leaf-level normalized difference vegetation index (NDVI), which is generally used to measure chlorophyll content by quantifying leaf greenness, and is closely related to fraction of absorbed photosynthetically active radiation (FPAR) [79, 80]. While not technically a functional trait (NDVI), traits based on spectral properties of leaves can be indicative of photosynthetic activity and plant stress, and from hereon, we include this complex trait as a functional trait for ease of discussion. A field spectroradiometer (ASD standard-resolution FieldSpec4, Malvern Panalytical) was used to measure leaf reflectance in the visible and reflected infrared spectral regions with 2,151 narrow bands (10 nm full width at half maximum) and 1 nm spacing between band centers. Measurements were made for three leaves using a leaf-clip attachment with its own light source and calibrated to % reflectance using data collected from a Spectralon white reference panel. Means for all bands among the three leaves were calculated for each individual tree. Specific wavelengths were used to estimate the modified red-edge NDVI. The modified red-edge NDVI was calculated using the following equation [81]:

$${\text{mND}}_{705}=\left({\text{R}}_{750}-{\text{R}}_{705}\right)/ \left( {\text{R}}_{750}+{\text{R}}_{705}-2\times {\text{R}}_{445}\right)$$

and was developed as an improvement to the standard NDVI to provide a more robust estimate of chlorophyll content [82] across a wide range of species and leaf structures [81]. Henceforth, this index will be referred to as “NDVI” in the text.

Specific leaf area (SLA) varies across global climate gradients [83], and high SLA values increase tree susceptibility to drought-induced mortality [84]. Specific leaf area (SLA) was measured on three fully matured leaves that were representative of the branch. After removing half of the petiole with a razor, the leaves were scanned into a computer using a Canon flatbed scanner (model # LiDE220) at 50 dpi. The leaves were then dried in an oven at 70 °C for 48 h and leaf mass was estimated on a digital scale with 1000th of a gram accuracy. SLA was calculated by dividing total leaf area by total leaf mass for all three leaves and averaged across the three leaves for a single SLA value for each individual tree.

### BLUP estimation

Best linear unbiased predictions (BLUP) were estimated for each trait to account for variation attributed to the design matrix and to increase trait accuracy because it anticipates regression of progeny to the mean observed [85]. Analysis was performed using ASreml Version 4.1 [86, 87]. Univariate variances were estimated within the framework of the linear mixed model:

$$\mathbf{Y}=\mathbf{X}\mathbf{b}+\mathbf{Z}{\varvec{u}}+\mathbf{e}$$

Where **Y** is the column vector of individual phenotypic values of the response variable, **X** is the design matrix associating observations with fixed effects, ***b*** is the vector of fixed effects, **Ζ** is the design matrix associating observations with random effects, ***u*** is a vector of random effects and **e** is the vector of residual errors assumed to be identically and independently normally distributed with *E*(*e*) = 0.

Two sets of analysis were conducted, the first at the family level for the purpose of checking the data for homoscedasticity and determining if there was a need for transformation, and the second at the individual tree level for the purpose of estimating BLUPs.

#### Univariate family model

Elements in ***b*** included the intercept and provenance effects while elements in ***u*** included replicate, row within replicate, column within replicate, plot and family. Residual plots were examined for homoscedasticity and appropriate transformations identified as outlined previously [86, 87]. The trait NDVI was log transformed, whereas the δ^13^C and SLA did not require transformation.

#### Univariate individual tree model

Elements in ***b*** and ***u*** were the same as for the univariate family model, with the exception that the family term was substituted with an individual tree, random additive effect. In this model, additive genetic covariance between relatives is modelled via the numerator relationship matrix. A one-tailed log likelihood ratio test with 0.5 degrees of freedom [87, 88] was used to test the significance of additive variance estimates for each trait.

### Reference genome

#### DNA extraction and sequencing

We isolated high molecular weight DNA suitable for long-read re-sequencing by following a nuclei and magnetic bead-based extraction protocol [89]. Briefly, 30 g of fresh leaf material from an individual from the Australian Botanic Gardens in Canberra Australia was processed with 150 ml nuclei isolation buffer using a high-powered blender. The homogenate was filtered using a funnel of Miracloth. Next, 100% Triton X-100 was added to extract the nuclei from chloroplasts. The nuclei pellet was washed twice with a chilled nuclei buffer. Nuclei pellet lysis was performed with a lysis buffer containing 3% Sodium dodecyl sulphate (SDS) followed by incubating at 50ºC. The DNA was cleaned of proteins by adding potassium acetate and pelleting. The supernatant was bound to Sera-MagTM SpeedBead magnetic carboxylate-modified particles (GE Healthcare). The beads were washed with 70% ethanol until clean. Size selection for fragments ≥ 30 kb was performed using a PippinHT (Sage Science, Beverly MA). MinION Mk1B was used to sequence the long-reads (Oxford Nanopore Technologies, ONT).

#### Nuclear genome assembly

Raw read libraries were filtered and trimmed in preparation of assembly with NanoPack [90] (NanoLyse version 1.1.0; NanoFilt version 2.6.0). First, ONT DNA control strand was removed. Next, 200 bp was trimmed from both 5' and 3' ends, removing sequencing adapters and low quality read ends. Finally, filtering removed all reads less than an average quality of 7 and less than 1 Kbp in length. Quality controlled read libraries were de novo assembled using the long read assembler Canu [91] (version 1.9; parameters: corOutCoverage = 200 "batOptions = -dg 3 -db 3 -dr 1 -ca 500 -cp 50", correctedErrorRate = 0.154, corMaxEvidenceErate = 0.15, -fast). Following assembly, contaminant contigs were identified with blastn [92] (version 2.9.0 +) using the NCBI nucleotide database [93] (versions BLASTDBv5). Identified contaminant contigs were removed with Blobtools [94] (version 1.1.1). Haplotigs, assembly artifacts, and plastid contigs were removed from assemblies with purge haplotigs [95] (version 1.1.0). Next, all assemblies were polished with the long read polisher Racon [96] (version 1.4.11) combined with minimap2 and the short read polisher Pilon [97] (version 1.23) combined with BWA-MEM [98]. Contigs of less than 1 Kbp were removed and manual curation of all remaining contigs was performed with MUMmer [99] (version 4.0.0beta2) to identify plastid DNA. Finally, our assemblies were scaffolded by RaGOO [100] using synteny information provided by the previously published *Eucalyptus grandis* genome [101]. Genome completeness was assessed with BUSCO [102] (version 3.0.2) and Lai [103] (version beta3.2). See Figure S1 for a summary of our assembly statistics.

#### Chloroplast assembly

Chloroplast reads were identified and subsequently extracted by aligning all reads with minimap2 [104] against a composite chloroplast genome made up of all published *Eucalyptus* chloroplast genomes. Chloroplast reads were identified within the alignment file with samtools v1.9 view [105] and extracted from all curated ONT reads using seqtk subseq (version: 1.3-r106; [106]). 1,000 chloroplast reads were randomly sampled using seqtk sample and assembled with Unicycler [107] (version 0.4.8) and polished using Pilon [97]. To confirm that the assembled contig was a chloroplast genome, an alignment dot plot was made of our chloroplast genome to the published *Eucalyptus* chloroplast genomes, using MUMmer [99].

#### Repeat and gene annotation

Prior to gene annotation repetitive regions in *C. calophylla’s* genome were identified and soft masked with RepeatMasker [108] (version 4.1.1) using de novo repeat libraries created with EDTA [109] (version 1.9.6). Protein-coding genes and transcripts were predicted by BRAKER2 [110] (version 2.1.5; parameters: epmode), using 306,675 proteins sequences from Myrtaceae (Taxonomy ID: 3931) and 371,118 proteins sequences from *Arabidopsis thaliana* (Taxonomy ID: 3702) obtained from the National Center for Biotechnology Information [93] as homology evidence.

### Library preparation & variant calling

DNA extraction for whole genome sequencing was performed by the Australian Genomic Research Facility (AGRF, Adelaide, SA Australia) using a modified CTAB method [111]. We generated short-read whole-genome shotgun DNA sequencing libraries using a low-cost transposase-based protocol [89]. Briefly, we quantified DNA concentrations using a fluorometric Quant-iT™ high sensitivity dsDNA assay kit (Molecular Probes™ Q33120). To normalise concentrations among samples, we diluted DNA to 2 ng/μl, quantified again and then diluted to 0.8 ng/μl. To form sequencing libraries, we combined 3 μl of each sample (approx 2.24 ng) with a small quantity of a Nextera™ tagment DNA enzyme (Illumina catalogue #15,027,865). To decrease costs, we performed this tagmentation reaction at 1/5th volume and 1/5th concentration of the manufacturer's protocol, i.e., 1/25th reactions. We amplified the libraries and added custom index sequences during 13 cycles of PCR. We purified and size-selected libraries using two SPRI-bead based cleanups and electrophoresis-based final size selection for insert sizes between 200 and 500 bp. We sequenced these libraries on a single S4 flow-cell on an Illumina NovoSeq 6000 instrument at Genomics West Australia/Telethon Kids Institute, Perth, West Australia.

Sequencing yielded between 3 and 10 Gbp per sample (~ 10-30X coverage), pooled across all sequencing runs (see Fig. 1 in main text). We discovered genetic variation among samples following a previous approach [39]. Briefly, we filtered, trimmed, and merged pairs of raw sequencing data using AdapterRemoval [112], then aligned reads to reference genomes using BWA-MEM version 0.7.15 [98, 113]. We detected short genomic variants using bcftools mpileup, normalised variants with bcftools norm, and performed initial variant filtering with bcftools filter [114]. Reads were aligned against our custom *Corymbia calophylla* reference genome. During initial variant filtering, we discarded variants with quality < 25, fewer than five reads in total across all alleles in all samples and fewer than three reads supporting the alternate allele across all samples. Resulting in 91 million pre-filtered single nucleotide variants, a variant every ~ 4 bp, which is normal among eucalypt species [39].

### Filtering

After variants were called using the above pipeline, additional filtering was performed in PLINK 2.0 [115] with the following thresholds. Minimum read-depth was set to six. We extracted biallelic variants only, to ensure all variants were biallelic and minimise complex signals. The minimum basepair distance between variants was set to 10. Minor allele frequency (MAF) was set to 0.01, to have sufficient power for GWAS detection. Missing data threshold was set to 0.5 but the average missing data in the data set was 0.2. Resulting in a dataset with 6.5 million SNPs across all 11 chromosomes.

### Linkage disequilibrium

Linkage disequilibrium (LD) was measured using median base pair distance to half-maximal *r*^2^ values using boringLD v0.3.0 (https://github.com/kdmurray91/ boringld). We set the window size to 30 kbp with a 15 kbp overlap. We fitted analytical models of the decay of *r*^2^ as a function of inter-SNP base pair distance using formulae derived by Hill and Weir [116] and then calculated base pair distance to half-maximal *r*^2^ for each window. We summarized per-window estimates of half-maximal *r*^2^ across all genome windows for a global *r*^2^ estimate. To test if LD was a function of the number of SNPs within each window, we used a linear model within each chromosome (Figure S2). The linear fit was significant for all chromosomes but the *r*^2^ values were low, this pattern was driven by the windows with very few SNPs. We also use LDSC to obtain LD scores which are the cumulative sum of r^2^ values across SNPs within 30 kb windows [117] and plotted these scores for each chromosome using ggplot2 [118] and R [119].

### Associations

Genome wide association studies (GWAS) were performed in Plink2 for each of the three functional traits. We used the individual BLUP estimates as the functional trait inputs, as this accounted for experimental site effects. We used the first 10 axes from an MDS as a covariate for population structure (first two axes are plotted and shown in Figure S3). We used the general linear model (glm) function to calculate *p*-values. We note that the power of GWAS analyses increases with higher genetic variation [120], but *Corymbia calophylla* is known to have extremely high diversity across its range with high connectivity [74, 121], making this species an exceptional study organism for this type of analysis. We also tested the associations of the GWAS using GEMMA with kinship matrix as a random covariable and the outputs resulted in nearly identical *p*-value distributions to the Plink2 analysis (Figure S6). We imported the Plink2 results to R [119] and adjusted the p-values for multiple comparisons using the Benjamini–Hochberg method (BH). Then used the *CMplot* command from the *CMplot* package to visualise the *p*-value distribution in a manhattan plot format. To ensure that the significant associations identified using the GWAS approach were not random, we used 100 permutations across the phenotype data in two ways and ran the Plink2 GLM analysis for both permutation variations. The first was completely random using the sample function in R without replacement, and the second was keeping the family structure of phenotypes and resampling among families. 

The qq plots suggest that the *p*-values were inflated regardless of the covariable used (population or family structure; Figure S6). Both GWAS methods identified very similar groupings of SNPs (51–65% total similarity and top 50 SNPs were 88% similar for δ^13^C). The differences between the distribution of BH adjusted *p*-values were significantly different between the real and permuted datasets (t.test: T_1,1.3 m_ = -2648.8, versus Random *p* < 0.001; T_1,1.3 m_ = -244.4, versus Family *p* < 0.001), suggesting that the adaptive variants were not due to chance. We wanted to determine if the associations between SNP and trait were associated with local genomic structure, as described in Li and Ralph [122]. Therefore, we used the package lostruct in R to investigate the structure within 1000 SNP windows (this is equivalent to approximately 10 kbp) within each chromosome, creating between 400 and 600 windows per chromosome. Then we compared the first two axes within chromosomes to the location of adaptive genomic regions for three major areas of association on chromosome 3, 8, and 10. We found that anomalous local population structure among 1000 SNP windows was not localised near regions that were significantly associated with phenotypes (Figure S7). Significant haplotype blocks within these three major regions were identified using Haploview [123] with 500 max kb, 0.05 minor allele frequency threshold, and block significance was determined using the default option of 95% confidence intervals [124].

### SNP heritability

To calculate SNP based heritability, we used the GEMMA model to describe the proportion of variance in phenotypes explained (PVE). GEMMA fits a univariate linear mixed model for marker association tests with a single phenotype, and for estimating the PVE by all variants [125]. We acknowledge that this work is performed in one common garden and that shared environments are known to inflate heritability [126]. However, upward bias due to shared environments would be consistent across populations [127] such that the heritability relationships are comparable among populations within the study species. This difference between actual and inflated heritability indicates that not all variation identified is adaptive, further quantitative experiments would need to be performed to confirm these results. We also determined genetic correlation (r_g_) between the three traits using LDSC [128], following the author’s recommendations.

### Epistasis & pleiotropy

We attempted to uncover some of the complex epistatic and pleiotropic relationships between variants and traits by use of the combined analysis of pleiotropy and epistasis (CAPE) package in R [129], which implements an analytical method described in Carter et al. [130] to explicitly test for these complex interactions. This method was designed for datasets that include populations with mixed genetic variation, and is therefore appropriate for our study design. CAPE calculates both the main effects, which are the effect of a SNP from the set of all pairwise regressions that included that SNP, and the directional influences of that SNP that interact epistatically. We used the following parameters for the CAPE analysis (parameter file available online): traits_scaled = true, pval_correction = fdr, alpha = 0.5, peak_density = 0.8, tolerance = 10, num_alleles_in_pairscan = 300, maxpair_cor = 0.5, pairscan_null_size = 1000. We used a high peak_density because of the quick LD decay, as suggested in the CAPE documentation. We also used a num_alleles_in_pairscan of 300 to limit the number of SNP pair analyses, this results in a different outcome for each run because we do not test all 104,329 pair possibilities. To be clear, individual SNP pair outcomes will not change, it is whether or not the individual SNP pair is randomly included in the output. Even so, the result shown here is a representative subset of these interactive effects. Both the inputs and outputs for our specific CAPE analysis are provided online, so the user can recreate our figures but also explore other individual runs and create new figures. 

### Functional annotations

The program snpEFF [131] was used to identify the location of significantly associated SNPs using the *Corymbia calophylla* genome (NCBI txid34324; assembly ASM1418284v1). Variants found within genes were recorded as synonymous or nonsynonymous, in addition variants in regulatory regions found within 5,000 base pairs of genes were recorded as being upstream or downstream, along with the number of base pairs between the gene and SNP. We recorded putative impact of the SNP on gene function and generally moderate effects are from nonsynonymous SNPs (changes in amino acids; ‘M’ in Table 1), low effects are from synonymous SNPs (no changes to amino acids; ‘L’ in Table 1), and high effects are from frame shifts or changes to start/stop codons (loss off function; ‘H’ in Table 1). We also specified which variants are in promoter regions, defined here as being within 500 bp upstream of the gene. Then, orthofinder was used to identify homologs between *C. calophylla* and *E. grandis* genomes [132], and assign putative functions to predicted genes identified as significant. We also provided results from a scale mapper to identify possible orthologs across KEG, COG, and eggNOG databases using eggnog-mapper [133] (version: 5.0) using the sequence aligner diamond [134] (version: 2.0.15).

### Supplementary Information

Additional file 1: Tables S1 – S5, S7. Figs S1 – S6.

Additional file 2: Table S6.

## Data Availability

The datasets generated and/or analysed during the current study are available in the European Variation Archive (EVA) repository, project code – PRJEB67343 (https://www.ebi.ac.uk/eva/?eva-study=PRJEB67343).
