# Supplementary material for: Genomic determinants, architecture, and constraints in drought-related traits in Corymbia calophylla
Source: BMC Genomics. 2024 Jun 27;25:640. doi: 10.1186/s12864-024-10531-8 (PMC11209971; doi:10.1186/s12864-024-10531-8)
Supplement: Supplementary file 1 — Additional file 1: Tables S1 – S5, S7. Figs S1 – S6. [file 12864_2024_10531_MOESM1_ESM.pdf]

Supplementary Materials for

Genomic determinants, architecture, and constraints in drought-related traits in *Corymbia calphylla*

Collin Ahrens[1,2,*], Kevin Murray[3], Richard Mazanec[4], Scott Ferguson[3], Ashley Jones[3], David Tissue[1], Margaret Byrne[4], Justin Borevitz[3], Paul Rymer[1]

[1] Hawkesbury Institute for the Environment, Western Sydney University, Richmond NSW Australia; [2]Cesar Australia, Brunswick VIC 3058 Australia;[3] Research School of Biology, Australian National University, Canberra, ACT Australia;[4] Department of Biodiversity, Conservation, and Attractions, Western Australia

Correspondence to: collinwahrens@gmail.com

The following PDF includes the following:

Table/Figure #	text	Title	page #
Table S1	main	Assembly statistics for *C. calophylla*.	2
Table S2	main	Linkage disequilibrium summary table.	3
Table S3	main	SNP independent linear models	4
Table S4	main	List of genes and functions significant for all three traits.	5
Table S5	main	Annotation summary	6
Table S6	main	All annotations from orthofinder and eggNOG	.csv
Table S7	main	Regulatory region summary	7
Figure S1	main	Summary histograms for the reference genome	8
Figure S2	main	Summary of LDscores	9
Figure S3	main	Population structure from MDS analysis	10
Figure S4	main	LD (r^2) between SNPs within trait and among traits	11
Figure S5	main	Full variant network output from the cape analysis	12
Figure S6	methods	Comparison between covariables in GWAS	13
Figure S7	methods	Local genome population structure	14

Table S1. Assembly statistics for *C. calophylla*.

	Un-scaffolded		Scaffolded	
**Genome assembly statistic**	**Length / % of genome**	**Number**	**Length / % of genome**	**Number**
**Total (Mbp)**	394.86	422	394.90	36
**Longest contig/scaffold (Kbp)**	7,649.18	---	48,491.33	---
**N50 (Kbp)**	1,980.23	---	39,866.58	---
**ESize**	2,476,596	---	37,436,745	---
**Genes**				42,234
**Simple repeats**			1.15%	---
**Transposons**			34.80%	---
**Chloroplast size (bp)**			158,591	---

Table S2. Linkage disequilibrium summary table. SE = standard error; SD = standard deviation; windows = the number of 30kbp overlapping windows per chromosome; nsnp_mean = the mean number of SNPs per window; nsnp_max = the maximum number of SNPs per window; nsnp_min = the minimum number of SNPs per window.

CHR	mean	median	max	min	SE	SD	windows	nsnp_mean	nsnp_max	nsnp_min
**1**	468.385	211.5	75858	3	44.953	1932.448	1856	483.248	1289	2
2	315.865	121	8710	3	12.218	604.158	2481	442.117	1637	2
3	460.888	118	41687	3	33.397	1716.621	2671	453.957	1596	2
4	464.103	174	28841	3	33.410	1425.703	1824	502.582	1464	3
5	288.582	81	12590	3	12.398	697.158	3183	488.427	1684	2
6	455.929	200	16219	3	17.011	895.644	2794	448.495	1449	2
7	345.852	88	43652	3	25.285	1296.446	2655	465.772	1406	2
8	423.481	148	27543	3	20.238	1147.665	3241	498.036	1883	3
9	516.830	191	35482	3	37.231	1545.422	1744	456.223	1231	2
10	597.287	230	28184	3	32.752	1395.707	1834	448.543	1453	4
11	740.037	214	338845	3	184.084	8064.035	1937	451.173	1513	2

Table S3. Results from independent linear models of gene trait interactions in *Corymbia calophylla* with SNP as the independent variable and phenotype as the dependent variable. The total variation explained among the top 10 SNPs in a single model. Bolded SNPs are found twice in the table. All R^2 values are adjusted and significant ($p < 0.0001$). *maf* = minor allele frequency. *es* = full model effect size. VE = proportion of variation explained. ***, **, * significance in the combined model < 0.001, 0.01, 0.05 respectively. † - dropped from the combined model due to giSNP (genetically indistinguishable SNP) or too similar to another SNP.

	$\delta^{13}C3$				SLA				NDVI		
Variant ID	R^2	*maf*	*es*	Variant ID	R^2	*maf*	*es*	Variant ID	R^2	*maf*	*es*
10:14288468[cc]	0.25**	0.165	-0.23 ±0.03	8:25569614[cc]	0.34***	0.497	-0.074 ±0.01	7:6588079[cc]	0.17	0.416	-0.011 ±0.002
3:38317350[cc]	0.24	0.174	-0.19 ±0.03	**11:24419468[cc]**	0.30***	0.135	0.093 ±0.01	8:25645984[cc]	0.12**	0.494	0.011 ±0.002
3:38370345[cc]	0.21**	0.160	-0.22 ±0.02	7:6588358[cc]	0.09*	0.119	0.074 ±0.01	7:20379383[cc]	0.11***	0.047	0.030 ±0.004
8:25630714[cc]†	0.21	0.160	-0.23 ±0.02	8:25649571[cc]	0.08	0.071	0.10 ±0.01	**11:24419468[cc]**	0.10	0.135	-0.017 ±0.002
8:25620946[cc]	0.21	0.169	-0.22 ±0.02	3:38327356[cc]	0.07	0.064	0.11 ±0.01	11:16551085[cc]	0.05	0.019	-0.041 ±0.006
8:25550760[cc]	0.19**	0.128	0.17 ±0.03	3:38388540[cc]	0.07	0.063	0.10 ±0.01	1:4110088[cc]	0.05	0.068	-0.025 ±0.003
3:38385682[cc]	0.18*	0.146	-0.21 ±0.02	10:14497305[cc]†	0.07	0.063	0.10 ±0.01	10:14265736[cc]	0.05*	0.396	0.013 ±0.002
3:38332167[cc]	0.18**	0.166	0.17 ±0.02	3:38272343[cc]†	0.07	0.063	0.10 ±0.01	10:14473331[cc]	0.05	0.427	0.013 ±0.002
10:14324927[cc]	0.18***	0.455	0.15 ±0.02	3:38275883[cc]†	0.07	0.063	0.10 ±0.01	3:38335476[cc]	0.05	0.400	0.014 ±0.002
9:17931039[cc]	0.18	0.084	-0.13 ±0.02	3:38321179[cc]†	0.07	0.063	0.10 ±0.01	3:38306212[cc]†	0.04	0.412	0.014 ±0.002
Combined 10 SNP VE	0.51***				0.48***				0.34***		
w/ epistatic interactions VE	0.63***				0.47***				0.40***		
Full model VE	0.55***				0.27***				0.66***		

Table S4. The 11 genes that were found to be significantly associated with all three traits are expressed during growth and development processes, including in plant structure such as guard cell and leaf structure (Eucgr.A00085, Eucgr.B03379, Eucgr.C00318, Eucgr.K02549, Eucgr.H02572, Eucgr.H02574, Eucgr.H02575 and Eucgr.C00581). Eucgr.A00280, Eucgr.C00467, Eucgr.C00296

Gene ID	Function
Eucgr.A00085	PF00226 - DnaJ domain;AT4G09350-NA|Chaperone DnaJ-domain superfamily protein|nucleus
Eucgr.A00280	PF00226 - DnaJ domain;AT4G09350-NA|Chaperone DnaJ-domain superfamily protein|nucleus
Eucgr.B03379	PTHR11516:SF4 - PYRUVATE DEHYDROGENASE E1 COMPONENT ALPHA SUBUNIT, MITOCHONDRIAL;AT1G59900-AT-E1 ALPHA,E1 ALPHA|pyruvate dehydrogenase complex E1 alpha subunit|mitochondrion
Eucgr.C00296	PF00560//PF00931//PF01582 - Leucine Rich Repeat // NB-ARC domain // TIR domain;AT1G27170-NA|transmembrane receptors;ATP binding|cytosol
Eucgr.C00318	PF00560//PF00931//PF01582 - Leucine Rich Repeat // NB-ARC domain // TIR domain;AT1G27170-NA|transmembrane receptors;ATP binding|cytosol
Eucgr.C00467	PF00560//PF00931//PF01582 - Leucine Rich Repeat // NB-ARC domain // TIR domain;AT1G27170-NA|transmembrane receptors;ATP binding|cytosol
Eucgr.C00581	PTHR10903//PTHR10903:SF23 - GTPASE, IMAP FAMILY MEMBER-RELATED // SUBFAMILY NOT NAMED;AT5G20300-NA|Avirulence induced gene (AIG1) family protein|cytosol
Eucgr.H02572	PTHR11260:SF16 - GLUTATHIONE S-TRANSFERASE;AT2G29420-ATGSTU7,GST25,GSTU7|glutathione S-transferase tau 7|cytosol
Eucgr.H02574	PTHR11260:SF16 - GLUTATHIONE S-TRANSFERASE;AT2G29420-ATGSTU7,GST25,GSTU7|glutathione S-transferase tau 7|cytosol
Eucgr.H02575	PTHR11260:SF16 - GLUTATHIONE S-TRANSFERASE;AT2G29450-AT103-1A,ATGSTU1,ATGSTU5,GSTU5|glutathione S-transferase tau 5|cytosol
Eucgr.K02549	PF04414 - D-aminoacyl-tRNA deacylase;AT2G03800-GEK1|D-aminoacyl-tRNA deacylases|cytosol

Table S5. Annotation summary at the chromosome level. Ts/Tv = transition vs transversion ratio; syn = synonymous; NS = nonsynononomous; up = upstream, within 5kbp of a gene; down = downstream, within 5kbp of a gene; high = high effect allele; mod = moderate effect allele; low = low effect allele.

Chr	Length	Variants	rate	Ts/Tv	syn	NS	up	down	high	mod	low
1	27,855,297	477,415	58	2.69	33,140	31,195	275,030	301,861	1,133	31,195	35,973
2	37,224,018	581,410	64	2.68	41,212	39,269	347,667	381,326	1,541	39,269	44,792
3	40,088,764	638,500	62	2.83	36,473	36,223	297,589	338,288	1,479	36,223	39,573
4	27,387,958	486,376	56	2.69	29,489	28,267	253,848	278,156	1,132	28,267	31,964
5	47,765,057	820,982	58	2.83	45,485	46,371	400,197	433,848	1,949	46,371	49,160
6	41,948,806	665,206	63	2.50	50,266	46,334	415,792	456,759	1,654	46,334	54,516
7	39,866,576	650,378	61	2.82	37,187	37,840	317,198	349,265	1,631	37,840	40,367
8	48,491,325	853,957	56	2.73	47,288	51,348	4,249,308	4,252,395	3,618	51,348	51,653
9	26,074,809	421,748	61	2.65	24,555	26,113	244,540	260,323	1,622	26,113	26,726
10	27,534,854	437,473	62	2.52	34,601	31,818	277,415	303,576	1,174	31,818	37,723
11	29,061,480	464,194	62	2.52	35,582	32,815	306,004	340,638	1,194	32,815	38,557

Table S7. Breakdown of significant variants within different distances from a gene.

		genic	500bp	5kb	10kb	50kb	total
d13C	Chr3	1	0	6	27	73	74
	Chr8	4	17	37	37	37	41
	Chr10	3	6	37	60	84	88
SLA	Chr3	0	0	1	6	17	17
	Chr8	0	5	13	13	13	13
	Chr10	0	0	8	10	15	16
NDVI	Chr3	0	0	2	9	23	23
	Chr8	0	4	16	16	16	16
	Chr10	0	0	11	17	25	26

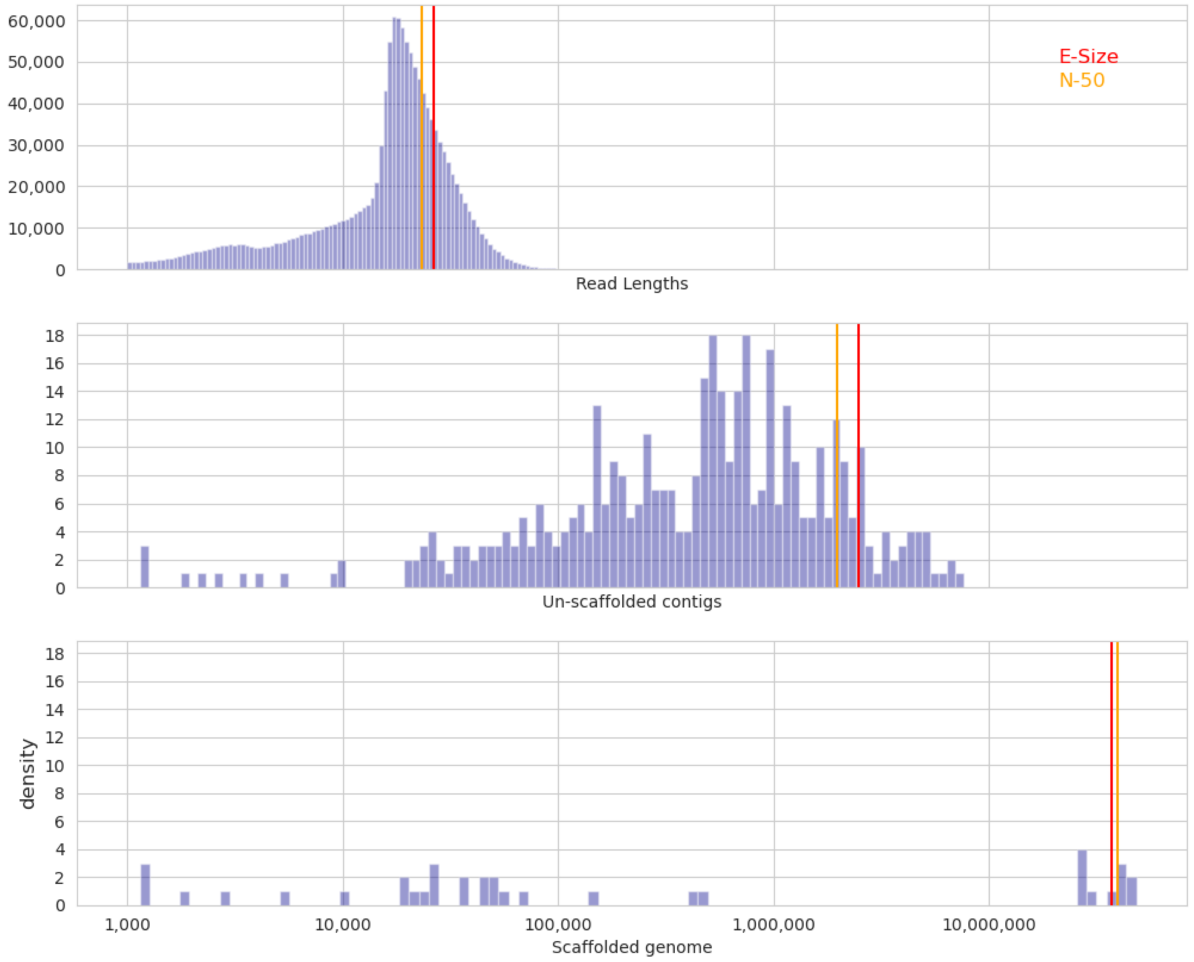

Figure S1. Summary histograms for the reference genome. Top is a histogram of read length. Middle is the filtered and haplotig purged assembly. The bottom histogram is the final scaffolded genome. Showing N50 (orange) and E-size (red) for each histogram.

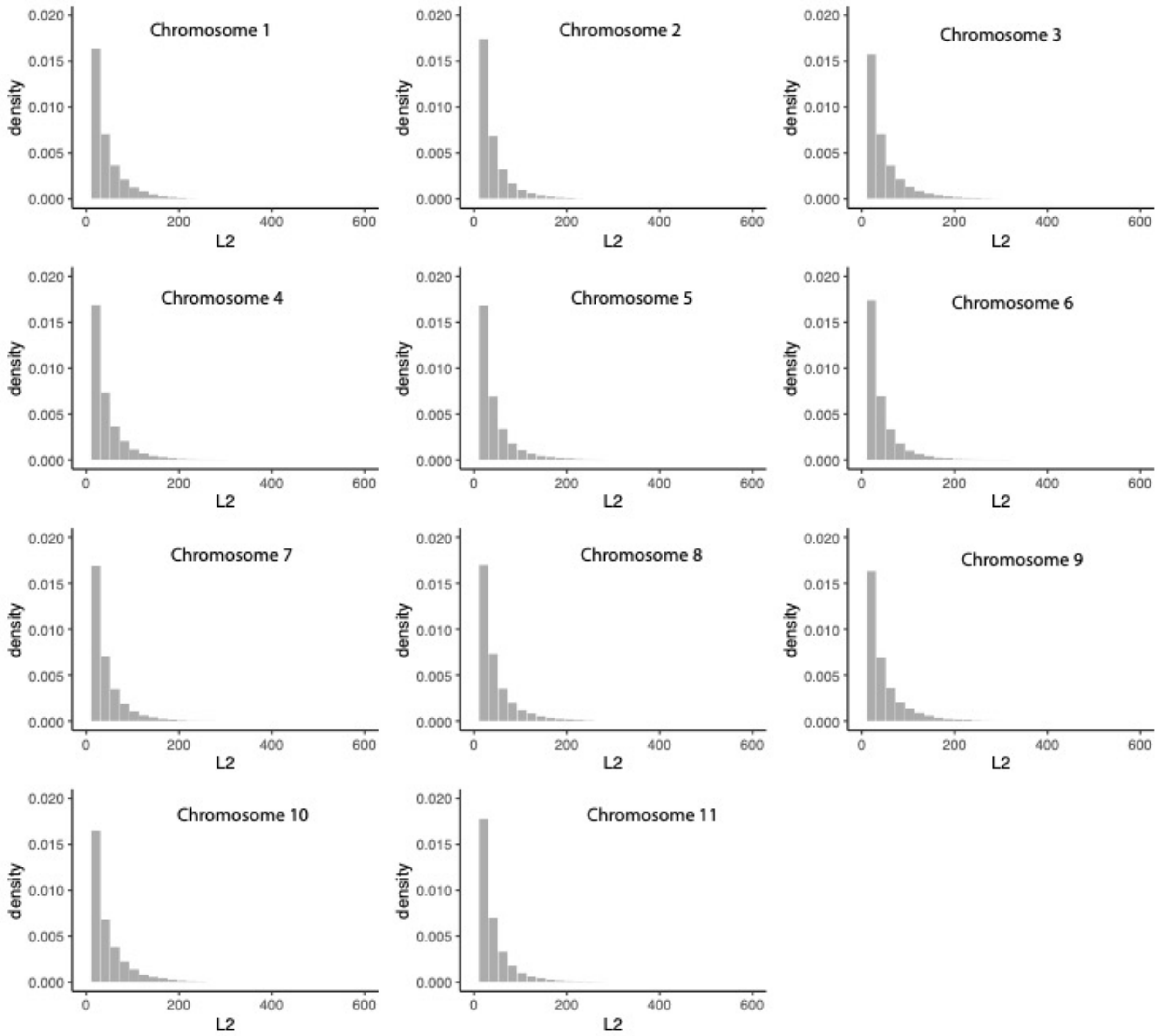

Figure S2. Summary histograms for LD scores (L2) for each chromosome. LD score is the sum of r2 for that variant across a 30kb window.

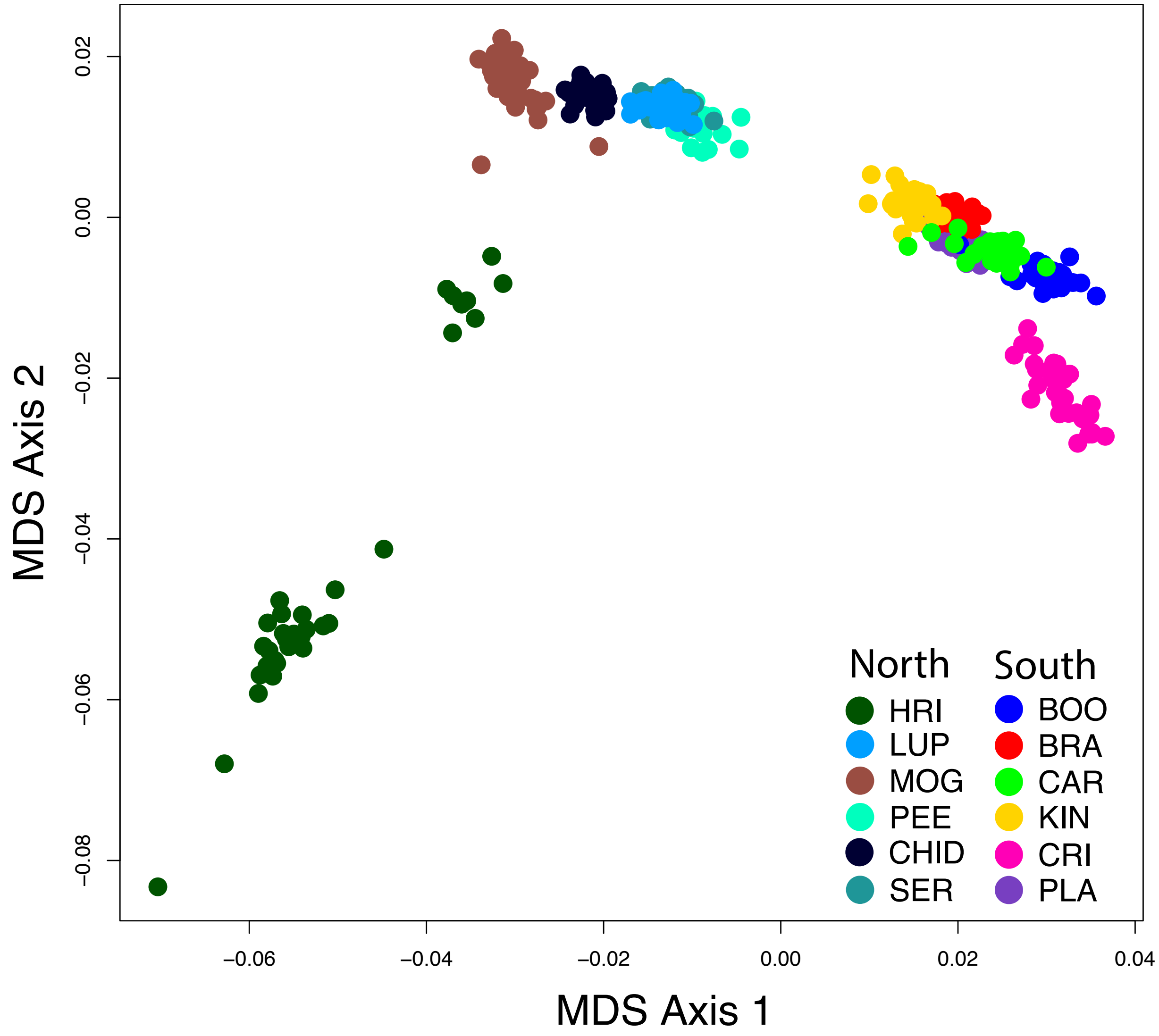

Figure S3. Population structure among all 456 individuals across 12 populations using the first two axes from the multidimensional scaling analyses that was used as a covariable in the GWAS analysis. Color represents population and there is a clear gap between the southern populations on the right and the northern populations on the left.

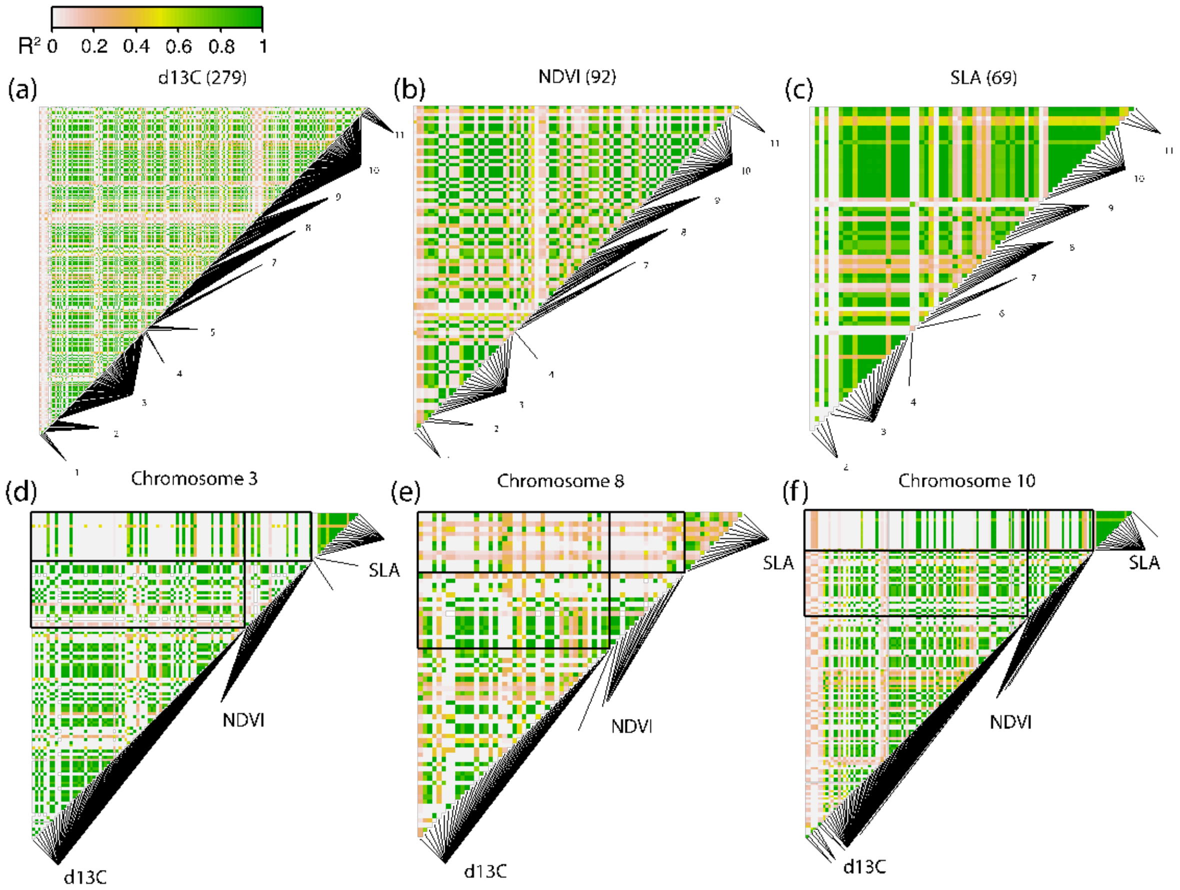

Figure S4. Linkage disequilibrium (r^2) between SNPs within trait (a-c), and among traits within chromosomes (d-f).

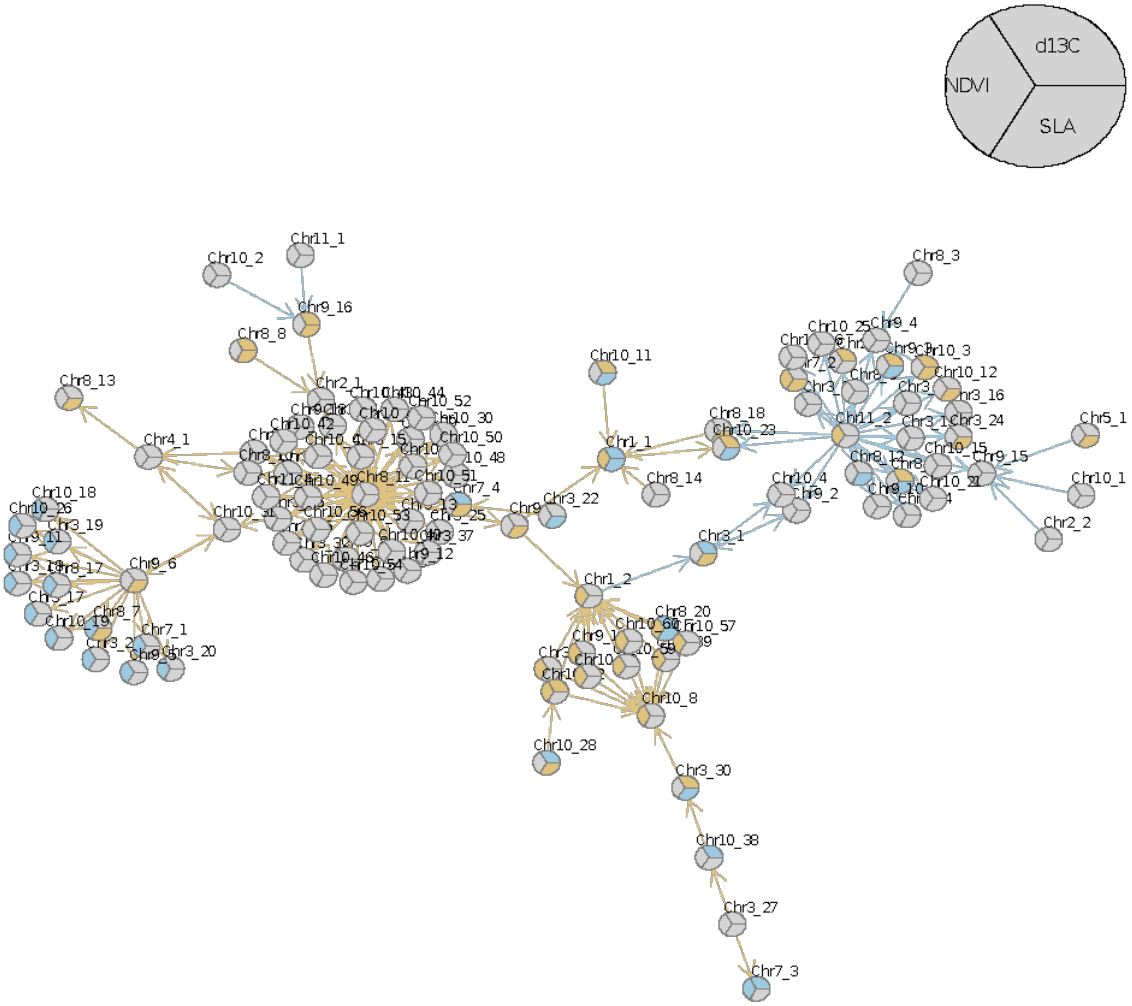

Figure S5. Full variant network output from the cape analysis. This view focuses on the structure of genetic interactions, regardless of variant position. Each node is one genetic SNP. Each is plotted as a pie chart with each trait as a specific piece of the pie. Significant effects are indicated by brown or blue section colouring, corresponding to positive (brown) or negative (blue) main effects with gray indicating no significant main effect. Interactions are shown as arrows between the nodes. Pie charts with both the brown and blue colours for different traits are indicative of antagonistic pleiotropy.

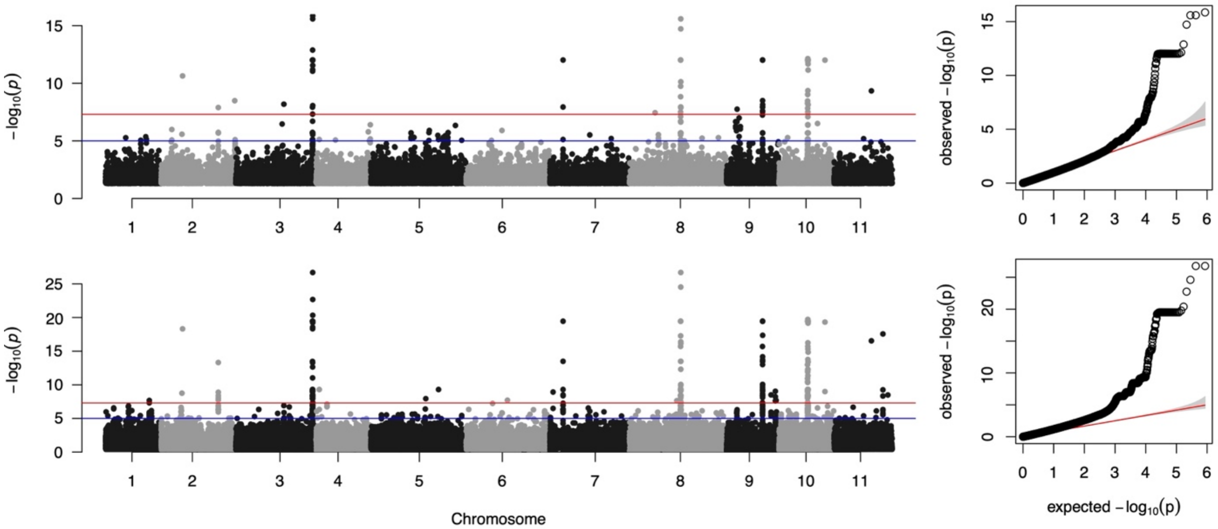

Figure S6. Manhattan plots from the $\delta^{13}C$ GWAS controlling for kinship matrix (top row) as a random variable and population structure (bottom row) as a fixed variable. Patterns remain nearly identical except for the lower p-values when using kinship matrix as the covariable. The correlation between these p-values was 0.56 calling nearly identical significant SNPs. These correlations support the robustness of the analyses.

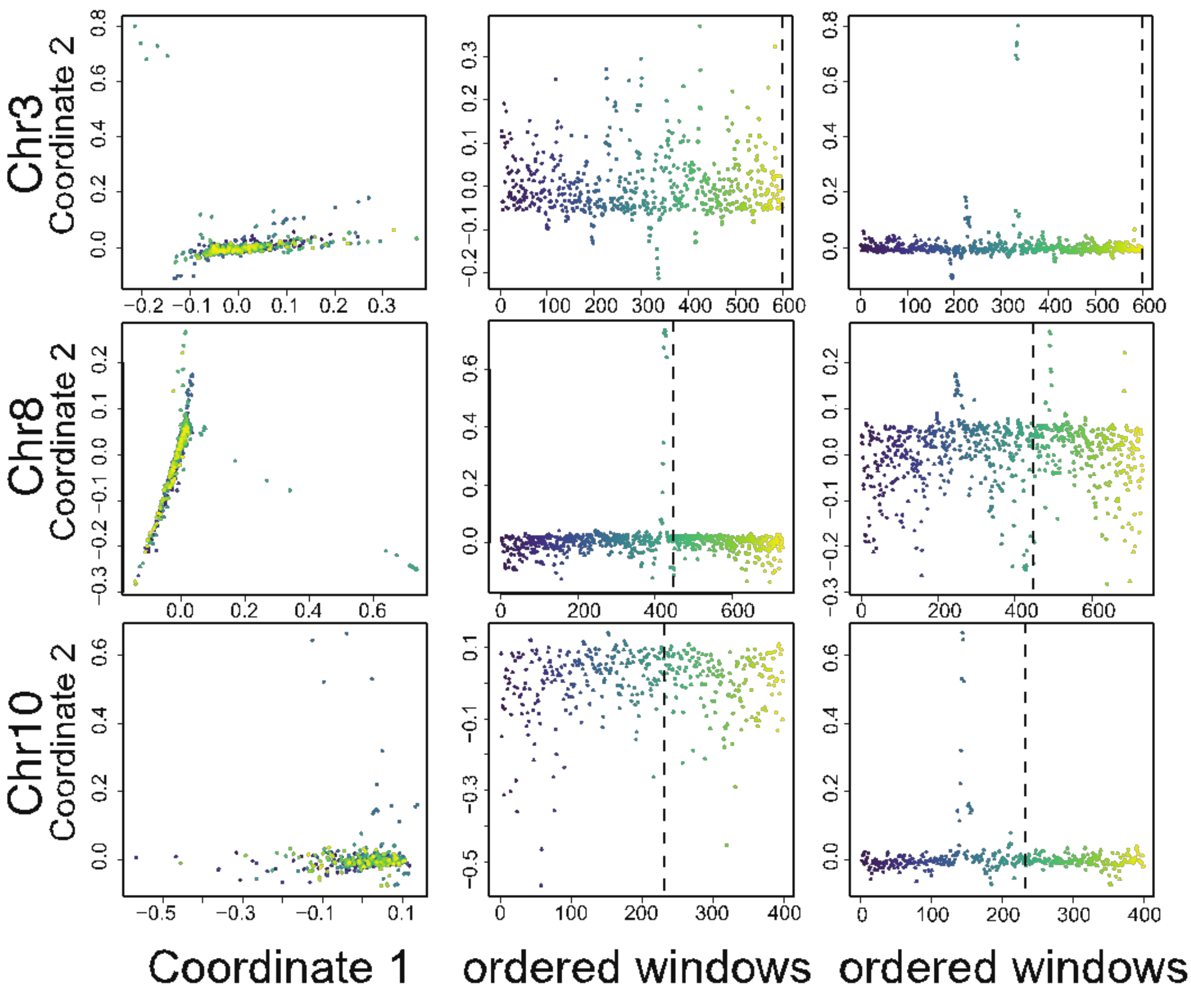

Figure S7. Local genome population structure for the three important chromosomes calculated in 1000 SNP windows. Column two is coordinate 1 (y-axis) version window number (x-axis) and column three is coordinate 2 (y-axis) versus window number. Dashed grey line indicates the window in which peaks with significantly associated *P*-values occur.
